# On the Accuracy of Factory-Calibrated Low-Cost Soil Water Content Sensors

**DOI:** 10.3390/s19143101

**Published:** 2019-07-13

**Authors:** Jesús María Domínguez-Niño, Heye Reemt Bogena, Johan Alexander Huisman, Bernd Schilling, Jaume Casadesús

**Affiliations:** 1 Program of Efficient Use of Water in Agriculture, Institute of Agrifood Research and Technology (IRTA), Parc de Gardeny (PCiTAL), Fruitcentre, 25003 Lleida, Spain; 2 Institute of Bio- and Geosciences, Agrosphere Institute (IBG-3), Forschungszentrum Jülich GmbH, 52425 Jülich, Germany

**Keywords:** soil water content, 10HS sensor, calibration, sensor variability, specific calibration, CRIM model

## Abstract

Soil water content (SWC) monitoring is often used to optimize agricultural irrigation. Commonly, capacitance sensors are used for this task. However, the factory calibrations have been often criticized for their limited accuracy. The aim of this paper is to test the degree of improvement of various sensor- and soil-specific calibration options compared to factory calibrations by taking the 10HS sensor as an example. To this end, a two-step sensor calibration was carried out. In the first step, the sensor response was related to dielectric permittivity using calibration in media with well-defined permittivity. The second step involved the establishment of a site-specific relationship between permittivity and soil water content using undisturbed soil samples and time domain reflectometry (TDR) measurements. Our results showed that a model, which considered the mean porosity and a fitted dielectric permittivity of the solid phase for each soil and depth, provided the best fit between bulk permittivity and SWC. Most importantly, it was found that the two-step calibration approach (RMSE: 1.03 vol.%) provided more accurate SWC estimates compared to the factory calibration (RMSE: 5.33 vol.%). Finally, we used these calibrations on data from drip-irrigated almond and apple orchards and compared the factory calibration with our two-step calibration approach.

## 1. Introduction

Efficient irrigation management is essential for reducing water consumption. To this end, real-time monitoring of soil water content (SWC) is essential to optimize the amount and timing of water irrigation [1,2]. Electromagnetic (EM) methods, such as time domain reflectometry (TDR) (e.g., Reference [3]) and capacitance sensors [4,5], are most commonly used for soil water content measurements at the point scale. Capacitance sensors are often preferred over TDR sensors, as they provide real-time SWC at a lower cost. In addition, they were shown to be reasonably robust and precise, and consume less energy compared to TDR sensors [6,7,8]. Both TDR and capacitance methods make use of the strong dependence of the soil dielectric permittivity on volumetric SWC. As the dielectric permittivity of liquid water is much higher than the dielectric permittivity of the other soil components, SWC is the principal factor governing the apparent soil permittivity [9]. However, other soil properties such as salinity and texture may cause dielectric losses and disturb the SWC measurements with EM sensors [10]. These dielectric losses depend on the frequency of the electric field generated by the sensors and are especially important for sensors that work at frequencies between 1 and 200 MHz [11]. In addition, capacitance sensors can show substantial sensor-to-sensor variability, which affects the accuracy of the soil water content measurements if this is not considered [12,13]. One solution to compensate for this effect would be to directly calibrate each sensor individually with soil samples [14]. However, this procedure is time consuming and thus often not viable in case of a high number of sensors [5,6]. Alternatively, a two-step calibration procedure can be used [10,15,16,17]. In a first step, a calibration between sensor response and permittivity is established for each of the sensors. In this step, media with well-known dielectric properties (referred to as reference permittivity), such as air, glass beads [18], and 2-isopropoxyethanol [19], are used. The advantages of using these reference media are: (i) the avoidance of air gaps and density variations, (ii) the possibility to separate sensor- and soil-specific effects, and (iii) the ability to quickly calibrate multiple sensors for a wide range of dielectric permittivity. In a second step, an appropriate relationship between permittivity and SWC needs to be established. One possibility is to use available empirical or semi-empirical models that relate permittivity and SWC [20,21]. To obtain more accurate SWC measurements, a site-specific calibration accounting for variations in key soil properties can also be established using a limited number of soil samples. Here, the use of TDR measurements should be preferred because of its ability to directly provide dielectric permittivity and the higher accuracy of the permittivity measurements.

In this study, we focused on the low-cost capacitance SWC sensor 10HS (METER Group Inc., Pullman, WA, USA, 2018). The main goal was to analyze whether it is worthwhile to perform sophisticated sensor- and soil-specific calibrations instead of using the factory calibration suggested by the manufacturer. To this end, we carried out sensor-specific calibrations with 10HS sensors using reference media with well-known dielectric properties and determined permittivity-SWC relationships using undisturbed soil samples and TDR measurements. The permittivity of the undisturbed samples was related to SWC taking into account different properties such as porosity and permittivity of the solid phase. We then compared the factory and the two-step calibration approach using (i) packed sand samples with known SWC in a laboratory experiments and (ii) SWC time series obtained at two test sites.

## 2. Materials and Methods

### 2.1. Sensor Technology

**Figure 1:**
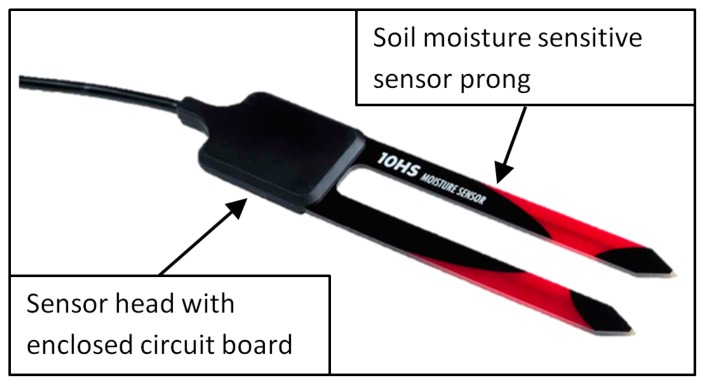
The 10HS sensor from METER Group Inc., USA.

In this study, we used the 10HS SWC sensor model manufactured by METER Group Inc., Pullman, WA, USA. This sensor has a prong length of 10.0 cm and a distance between the prongs of 2.2 cm (Figure 1). The 10HS sensor determines SWC using the capacitance method. According to the manufacturer, it has a probing volume of about 1 dm^3^, which is much larger than other low-cost SWC sensors like the EC-5 and EC-20, which have a volume of influence of 0.3 dm^3^ [13].

The 10HS sensor determines SWC by measuring the charge time of a capacitor (i.e., the soil-probe system), which is related to the permittivity of the soil surrounding the sensor [17]. The manufacturer provides a factory calibration to obtain SWC from the sensor response:

SWC (vol.%) = (1.16 × 10^−9^ (RAW^3^) − 3.95 × 10^−6^ (RAW^2^) + 4.89 × 10^−3^ (RAW) − 1.92) × 100 (1)

where RAW is the raw sensor count. According to the manufacturer, this calibration equation is valid for SWC in the range between 0 and 57 vol.%. Like all ECH_2_O SWC sensors of the METER Group, the 10HS sensor uses an oscillation frequency of 70 MHz. Therefore, the SWC measurements with the 10HS sensor may be affected by temperature and soil bulk electrical conductivity variations [13].

### 2.2. Study Area

We used 16 10HS sensors to measure SWC in an almond and apple orchard located in Menàrguens and Mollerussa (Lleida, Spain), respectively. Both orchards were equipped with a drip irrigation system. The almond plants were planted in ridges of 200 cm width and 50 cm height on top of the original soil. The soil material used to create the ridges consisted of a mixture of local soil and an organic amendment. The irrigation system of the almond orchard was located on the top of the ridge and consisted of a double tube system separated by 40 cm with drippers spaced at 100 cm intervals. The sensors were installed in the middle of the ridge at depths of 20 and 50 cm. The apple orchard had a single tube system with drippers spaced every 60 cm and the sensors were located under and between the drippers at depths of 15 and 30 cm. The properties of the soils are summarized in Table 1.

### 2.3. Laboratory Experiments

In this study, we relied on a two-step calibration approach to relate sensor response to SWC. In a first step, the relationship between sensor response and permittivity was established for each sensor (i.e., a sensor-specific calibration). In a second step, a site-specific relationship between permittivity and soil water content was developed using a limited number of soil samples using the TDR method (soil-specific calibration).

#### 2.3.1. Sensor Response—Permittivity Calibration for the 10HS Sensor

For the first calibration step, we used the approach of Bogena et al. [5] and calibrated 16 sensors. We used five calibration standards for sensor calibration (air, glass beads, and three mixtures of 2-isopropoxyethanol (i-C3E1) and deionized water with a defined volume fraction of i-C3E1). The properties of these reference media are described in Table 2. We used soda lime glass beads (type: Silibeads 4501, Sigmund Lindner GmBH, Warmensteinach, Germany) with a grain size between 0.25 and 0.50 mm and a dielectric permittivity of 3.34 [18]. The sensor response for all reference media was measured at 25 °C using the ProCheck device (Meter Group Inc., Pullman, WA, USA). The permittivity range from 1.0 to 34.8 covers most of the dielectric permittivity values found in natural soils. Table 2 shows that there is a considerable gap between 4 and 32%. Initially, pure I-C3E1 with an equivalent water content of 24% was also considered. However, pure I-C3E1 is highly hydrophilic. Therefore, these measurements were unreliable and not stable during calibration. For this reason, we decided to discard this solution from the analysis. Future studies should investigate alternative reference liquids to fill this gap.

Each of the 16 sensors was calibrated taking into account two immersions variants. In the first variant, only the sensor prongs were inserted in the reference media (i.e., incomplete immersion). In the second variant, the entire sensor including the sensor head with the electronics (see Figure 1) was fully immersed in the reference media. This second variant mimics a typical field installation where the sensor head is fully surrounded by soil, whereas the first variant represents a typical situation for crops planted in bags of growing media and laboratory SWC measurements. If the sensor electronics in the sensor head are not influenced by the permittivity of the surrounding media, both variants should provide the same sensor reading for a given dielectric permittivity.

Several precautions were considered to obtain precise calibrations. First, we used sufficiently large (6.4 dm^3^) polyethylene bottles (diameter of 19.5 cm, height of 23.0 cm) to fully include the sensing volume of the 10HS sensor (1 dm^3^). Second, the sensor was fixed and centrally immersed in the reference media to reduce the effects of sensor position on the measurements. Finally, possible degrading effects of the reference media on the plastic body of the sensor were minimized by carefully cleaning the sensor after each measurement and minimizing the contact time. The calibration station consisted of four plastic bottles containing the different dielectric reference media arranged on a workbench. The bottle with the glass beads was placed on a vibration machine in order to maintain the same packing density and not affect the calibration of the 10HS sensors. The other three bottles were placed on magnetic stirring devices to avoid demixing of the reference media. They were also covered with a lid to prevent evaporation. In addition, a bottle of water was used to clean the sensors after each measurement. Bogena et al. [5] provided a more detailed description of the set-up of the calibration workbench.

The sensor response (*ν*) was related to the dielectric permittivity (*K_a_*) using an empirical sensor response permittivity (SRP) model. In this study, the sensor response was related to the apparent dielectric permittivity using the following empirical model:

(2) $$K_{a}=\;\gamma \;+\;1\;/\;(\alpha \;+\;\beta \;/\;\nu )$$

where *ν* was the sensor response (voltage, V) and α, β and γ were fitting parameters. The RMSE between the predicted *K_a_* and the reference permittivity, ε_ref_, was used to express the accuracy of the SRP model. Empirical SRP models were already successfully applied to relate sensor readings of low-cost sensors to dielectric permittivity in several studies to account for sensor-to-sensor variability of various SWC sensors [6,22]. In addition, we investigate the decrease in accuracy when using a universal SRP model that ignores sensor-to-sensor variability of the 10HS sensors.

#### 2.3.2. Permittivity-Soil Water Content Relationships

To obtain soil-specific relationships between dielectric permittivity and SWC for the Menàrguens and Mollerussa test sites, we took 16 undisturbed samples using Kopecky rings with a length of 7.7 cm and a diameter of 5 cm. We took 4 samples at 20 cm depth and 4 samples at 50 cm depth from the Menàrguens test site and 4 samples at 15 cm and 4 samples at 30 cm depth from the Mollerussa test site. In the laboratory, we saturated the samples with deionized water and let them evaporate at room temperature. The volumetric SWC was determined twice a day from the weight of the sample, the known sample volume and the dry weight of sample determined at the end of the experiment by oven drying (65 °C, 48 h). The apparent dielectric permittivity of each sample was determined from measurements with a CS 640-L 3-rod TDR probe attached to a TDR-100 device (Campbell Scientific Inc., Logan, UT, USA). We used the internal TDR-100 algorithm to analyze the TDR measurements. One sample had to be discarded because shrinkage caused a significant decrease in volume. Therefore, the final data set consisted of dielectric permittivity and SWC measurements for 15 soil samples with known bulk density and porosity as provided in Table 3.

It should be noted that there is a difference in operating frequency between the 10HS sensor (70 MHz) and the effective frequency of TDR (100 to 500 MHz), although the latter is poorly defined and depends on the measurement set-up and TDR waveform analysis approach [23]. For the low-salinity and loamy soils investigated here, it is assumed that the operating frequency of 70 MHz for the capacitance sensors is sufficiently high to avoid effects of low-frequency polarization losses [24]. Therefore, the difference in measured apparent permittivity between the capacitance sensors and TDR is expected to be low. The alternative approach where 10HS sensors are used for soil-specific calibration would overcome possible differences in frequency, but has the disadvantage that sensor- and soil-specific calibration are convoluted. Therefore, we prefer not to use this latter approach.

Five empirical and semi-theoretical model variants were evaluated using the root mean square error (RMSE) between measured and predicted SWC. The first model was the empirical Topp model [20]:

(3) $$\mathrm{SWC}\;(\mathrm{vol}.\%)\;=\;(-5{.3\;\times \;10}^{-2}\;+\;2{.92\;\times \;10}^{-2}{\times \;K}_{a}\;-\;5{.5\;\times \;10}^{-4}{\;\times \;K}_{a}^{2}\;+\;4{.3\;\times \;10}^{-6}{\times \;K}_{a}^{3})\;\times \;100$$

In addition, we used four different variants of the complex refractive index model (CRIM) [25]:

(4) $$\mathrm{SWC}\;(\mathrm{vol}.\%)\;=\;100\;\times \;\frac{K_{a}^{\beta }\;-\;(1\;-{\;\eta )\;\times \;K}_{s}^{\beta }\;-{\;\eta K}_{air}^{\beta }}{K_{w}{\left(T\right)}^{\beta }\;-\;K_{air}^{\beta }}$$

where *K_a_* is the measured apparent dielectric sensor permittivity, *K_s_* is the dielectric permittivity of the solid phase, and *η* is the porosity. The value of the shape factor *β* was set to 0.5 [26]. The dielectric permittivity of air, *K_air_*, was assumed to be 1 and the temperature dependent dielectric permittivity of water, *K_w_*, was assumed to be 78.54 at 25 °C [27]. In the first variant (CRIM-1), we used the averaged measured porosity for all samples (*η* = 43%) and assumed that the dielectric permittivity of the solid phase, *K_s_*, was 4.4 based on the value for quartz [28]. In the second variant (CRIM-2), we again used the measured average porosity (*η* = 43%) but fitted *K_s_* to the data. In the third variant (CRIM-3), we used the mean porosity per soil and depth (Menàrguens: *η* = 48% and 36% at 15 cm and 50 cm depth, respectively; Mollerussa: *η* = 44% and 43% at 15 cm and 30 cm depth, respectively) and fitted *K*_s_ again. In the fourth and final variant (CRIM-4), we again used the mean porosity per soil and depth and now fitted the dielectric permittivity of the solid phase for each soil and depth.

#### 2.3.3. Sandbox Experiment

**Figure 2:**
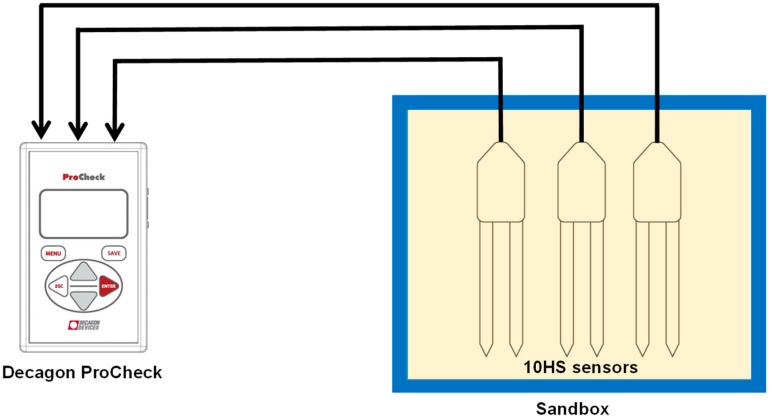
Top view of the sandbox experiment with three10HS sensor connected to Decagon ProCheck device.

In order to compare the accuracy of the factory calibration provided by the manufacturer with the two-step calibration developed in this study, a sandbox experiment was performed. The experiment was carried out in a box (length: 36.8 cm, width: 26.7 cm, height: 17.2 cm) which was filled with 15 kg of quartz sand, with a grain size diameter between 0.1 and 0.4 mm (F32, Quartzwerke, Frechen, Germany). To cover soil water contents between 0 and 35 vol. %, we added 0.5 dm^3^ of demineralized water in seven steps. Each time, the sand was thoroughly mixed with a blender before refilling into the box to achieve best possible soil homogeneity. During the refilling process, the soil material was carefully compacted to achieve similar soil density. The sand height and weight were measured to determine soil volume and soil density. Three 10HS sensors were installed in the central part of the box (see Figure 2) ensuring that the measurements were only affected by the sand inside the box. The ProCheck device (Meter Group Inc., Pullman, WA, USA) was used to determine the raw sensor response and the soil water content based on the factory calibration. For the two-step calibration, the universal SRP model determined using the reference media was used to convert the sensor response to permittivity, and the Topp equation [20] was used to determine SWC from permittivity. Additional corrections for the effect of electrical conductivity and temperature were not required here because of the use of demineralized water with low conductivity and the controlled temperature during the experiment.

## 3. Results and Discussion

### 3.1. Sensor Response—Permittivity (SRP) Calibration for the 10HS Sensor

The statistical results of the sensor response measurements in the different reference media for the two immersion variants are summarized in Table 4. It can be seen that mean sensor response increased with increasing permittivity and that the 10HS sensors showed considerable sensor-to-sensor variability as indicated by the average standard deviation and coefficient of variation of 13.8 mV and 1.41 %, respectively. This sensor-to-sensor variability is the consequence of intrinsic factors, such as subtle variations in the electrical components and probe geometry affecting the electromagnetic wave propagation characteristics [6]. It can also be seen that the sensor readings are affected by the immersion depth of the sensor into the reference media. Our experimental results indicate that with increasing permittivity, the 10HS sensor becomes increasingly affected by the depth of immersion. For the reference media M1-M3, only minor differences were found. However, significant differences were found for M4 and M5 with differences in mean sensor response of 0.02 V and 0.05 V, respectively.

**Figure 3:**
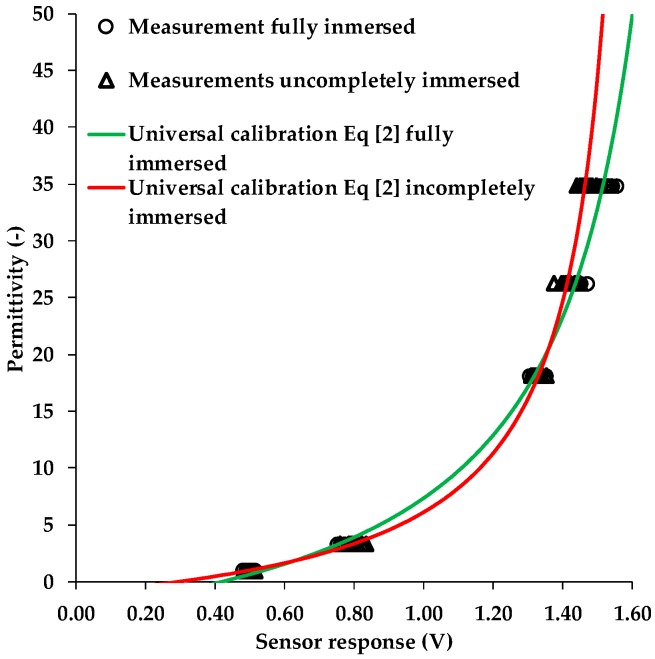
The response of 16 10HS sensors in five reference media for the two cases “incompletely immersed” and “fully immersed” as well as the corresponding universal Sensor Response–Permittivity (SRP) models.

In order to test how the differences in sensor response of the two immersion variants affect the sensor calibration, we fitted the SRP model (Equation (2)) to both calibration data sets. The fitted SRP models are presented in Figure 3 and the fitting parameters and the associated RMSE are provided in Table 5.

The difference between the two SRP models is clearly visible in Figure 3. In the case of incomplete immersion in the reference media, the slope is much steeper in wet soil. This has the following implications for the measurement accuracy of the 10HS sensor. First, the use of a calibration strategy based on incomplete immersion will overestimate permittivity in the range between 0.20–0.65 V and 1.35–160 V and will underestimate it in the range between 0.65–1.35 V ranges when the sensor is completely buried in the soil during the field experiments. Second, the sensor reading is less sensitive to changes SWC in the range between 1.35–1.60 V due to the steeper slope. In our field experiment, the 10HS sensors were completely buried in the soil. Therefore, we prefer the SRP model obtained from the fully immersed calibration data to describe sensor response-permittivity relationship of the 10HS sensors in the following.

### 3.2. Universal Versus Sensor—Specific Calibration

**Figure 4:**
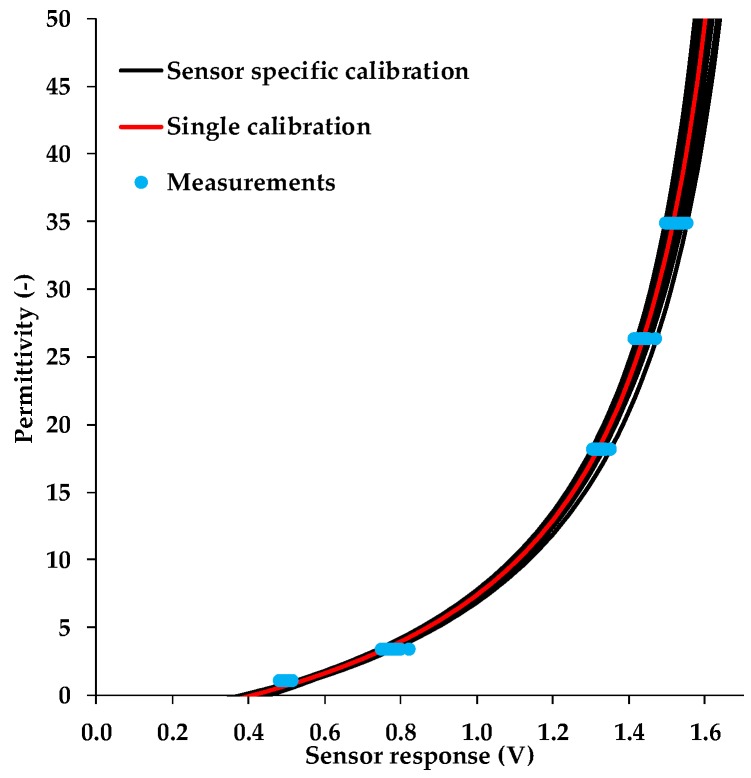
Universal Sensor Response–Permittivity (SRP) model fitted to the whole data set of every sensor and sensor-specific SRP models fitted to the sensor response measurement of each 10HS sensor.

The 10HS sensor exhibited considerable sensor-to-sensor variability (Figure 3). Therefore, we tested to which degree the 10HS sensors would benefit from a sensor-specific calibration. The comparison between universal and sensor-specific calibration of each of 16 sensors is presented in Figure 4. The RMSE between the reference permittivity (Table 2) and the apparent dielectric permittivity estimated using the fitted SRP model (fully immersed case) was used to evaluate to what extent a sensor-specific calibration could improve the accuracy of the permittivity estimates (Table 6). To put the results into perspective, the permittivity was converted to equivalent SWC using the Topp model [20].

In case a universal calibration function was used to relate sensor response to permittivity, the RMSE between estimated and reference permittivity increased considerably with increasing medium permittivity. The overall RMSE for *K_a_* determined using a universal calibration function was 1.421 (*θ_eq_*: 1.213 vol. %). Rosenbaum et al. [6] obtained similar RMSE values of 1.5 and 1.2 for the EC-5 and 5TE sensors (METER Group Inc., Pullman, WA, USA), respectively. Bogena et al. [5] found lower errors (RMSE *K*_a_: ~0.87, RMSE (*θ_eq_*: ~0.95 vol. %) for the low-cost SMT100 sensor (Truebner GmbH, Neustadt, Germany).

The use of sensor-specific calibration decreased the overall RMSE of *K_a_* to 0.427 *(θ_eq_*: ~0.642 vol. %). Sakaki et al. [12] obtained a similar accuracy for dry sand (±0.5 vol. %) and a lower accuracy for saturated sand (±2.8 vol. %) in case of the EC-5 sensor. Rosenbaum et al. [6] also investigated the EC-5 sensor, and found a lower accuracy for a sensor-specific calibration (~0.8, 1.4 vol. %). Finally, Qu et al. [22] investigated sensor-specific calibration for the SPADE sensor (Sceme.de, Horn-Bad Meinberg, Germany), and obtained a higher accuracy of 0.226 (0.4 vol. %). Given the standardized calibration process that reduced side effects such as variations in glass beads density as well as medium contamination to a minimum [5], we attribute the observed differences mainly to sensor-to-sensor variability which has been often observed for this kind of low-cost SWC sensors [6].

### 3.3. Permittivity–Soil Water Content Relationships

**Figure 5:**
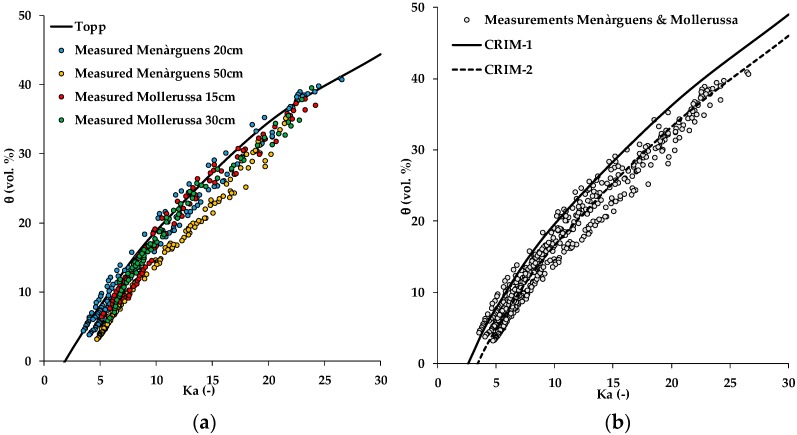
Relationship between apparent dielectric permittivity (*K_a_*) and soil water content for (**a**) all samples from the Menàrguens (20 cm and 50 cm depth) and (**b**) Mollerussa (15 cm and 30 cm depth) test sites and the fit of Topp model [20] and the complex refractive index models (CRIM-1) and (CRIM-2).

The relationship between the apparent dielectric permittivity and SWC of 15 undisturbed soil samples from Menàrguens (20 cm and 50 cm depth) and Mollerussa (15 cm and 30 cm depth) is shown in Figure 5a. It can be observed that the data were slightly different depending on location and depth. In a first step, the accuracy of the relationship proposed by Topp et al. [20] was evaluated. This empirical relationship resulted in a RMSE of 2.94 vol.% (Figure 5a, Table 7), which indicates a reasonably good match considering that the Topp model [20] is a “universal function” derived from experiments with limited variation in soil properties.

The first variant of the CRIM model (CRIM-1) considered the average porosity for all samples (43%) and a literature value for *K_s_* (4.40 for quartz [28]). The fit to the data is shown in Figure 5b and the resulting RMSE was 3.54 vol. % (Table 7). This indicates that the use of the CRIM-1 model based on measured average porosity and literature values for the permittivity of the solid phase resulted in a somewhat lower accuracy than the Topp model [20]. In the next variant (CRIM-2), *K_s_* was fitted. This resulted in a *K_s_* value of 6.3 and a better fit to the data (Figure 5) with a RMSE of 1.90 vol. % (Table 7). The fitted *K_s_* obtained with the CRIM-2 model is higher than that of the CRIM-1 model, which was based on the permittivity of quartz. Since most clay minerals have a higher permittivity than quartz [28], this is not surprising considering the relatively high silt and clay fraction of the Menàrguens and Mollerussa test site (see Table 1).

**Figure 6:**
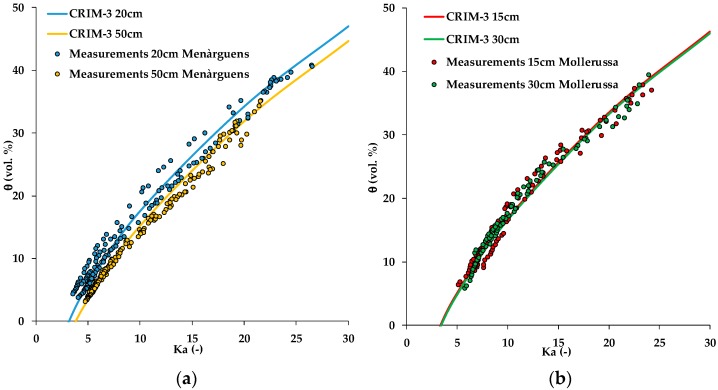
Apparent dielectric permittivity (*K_a_*)-soil water content for (**a**) all samples of the Menàrguens (20 cm and 50 cm depth) and (**b**) Mollerussa (15 cm and 30 cm depth) test sites and the derived complex refractive index model (CRIM-3) model.

In the next variant (CRIM-3), the variability in measured porosity was also considered. To this end, we averaged the porosity measurements per site and depth, resulting in a porosity of 48% and 36% for 20 and 50 cm depth, respectively, for the Menàrguens site and a porosity of 44% and 48% for 15 and 30 cm depth, respectively, for the Mollerussa site. Again, a single value of *K_s_* was fitted to the data, and this resulted in a *K_s_* value of 6.3. The RMSE further decreased to 1.43 vol.%, indicating that porosity is an additional control of the apparent dielectric permittivity-soil water content relationship. Figure 6 shows the fit of the CRIM-3 model to the experimental data. For the Menàrguens site, the CRIM-3 model was significantly different for 20 and 50 cm depth due to the different porosity. At 50 cm depth, there was a zone of larger compaction due to the transition between the ridge and the original soil. Therefore, the undisturbed soil samples showed higher bulk density and lower porosity. For the Mollerussa site, the CRIM-3 model predictions were similar for 15 and 30 cm depth, since the bulk density and porosity of both depths were similar.

**Figure 7:**
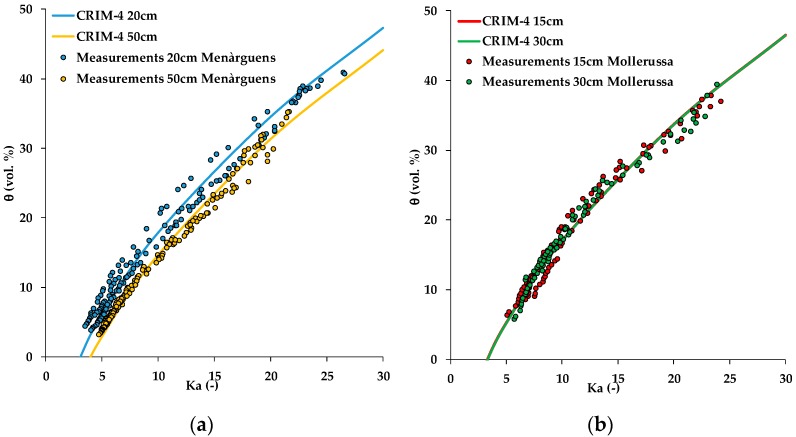
Apparent dielectric permittivity (*K_a_*)-soil water content for (**a**) all samples of Menàrguens (20 cm and 50 cm depth) and (**b**) Mollerussa (15 cm and 30 cm depth) test sites and the derived *K_a_-θ* complex refractive index (CRIM-4) model.

For the final variant (CRIM-4), both the dielectric permittivity of the solid phase (*K_s_*) and average porosity varied per depth and site (Figure 7). In this variant, the RMSE further improved to 1.37 vol.%, although the improvement was only subtle compared to the variant CRIM-3 with only a single value for *K_s_.* The fitted values for *K_s_* are given in Table 8, and varied in a small range only. In comparison to other studies, this fit is excellent. For instance, Robinson et al. [15], who evaluated the performance of several capacitive sensors including the Wet2 (Delta-T Devices), 5TE and 10HS sensors in well-characterized soils with variable texture, obtained accuracies that varied from 3.4 to 7.3 vol.%. Similar applications of the CRIM model by Rosenbaum et al. [29] and Qu et al. [30] resulted in RMSE values of 2.9 vol.% and 2.2–2.8 vol.%, respectively.

### 3.4. Comparison of Factory and Two-Step Calibration Approach

**Figure 8:**
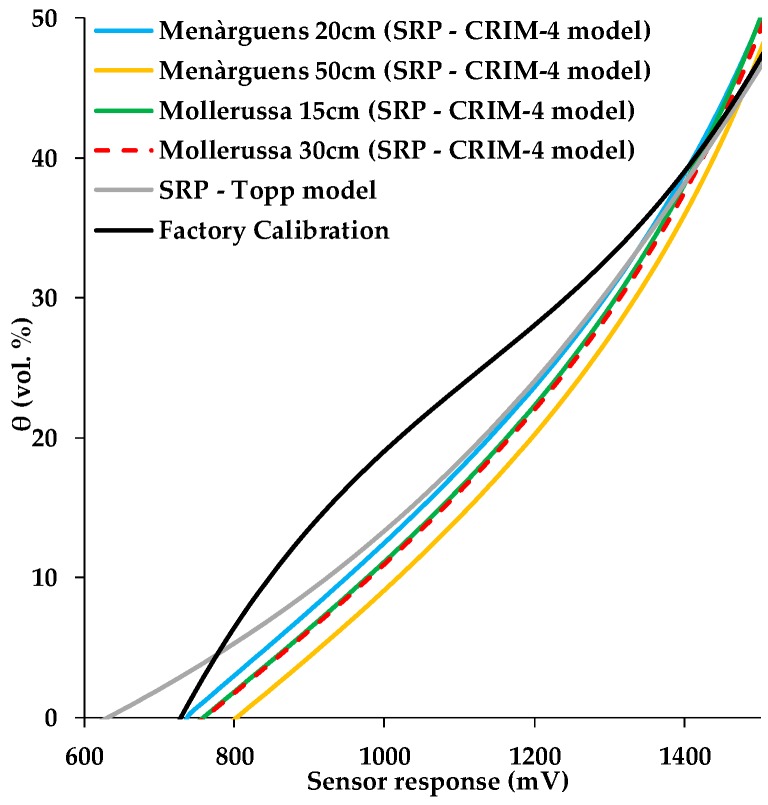
Comparison of the factory calibration and calibration curves for 10HS sensors obtained using the reference two-step calibration for soils samples from the Menàrguens and Mollerussa test sites. A calibration using the universal Sensor Response–Permittivity (SRP) model combined with the *K_a_-θ* Topp model [20] is also presented.

In order to compare the two-step-calibration approach with the factory calibration, we combined both calibration steps (SRP and CRIM model) to obtain a sensor response-SWC relationship. In the following, we consider the calibration approach using the SRP model for a fully immersed sensor head and the CRIM-4 variant as the “reference” two-step calibration. Figure 8 shows that there was a substantial difference between factory calibration and the “reference” two-step calibration as well as the calibration variant using a combination of the SRP model and the Topp equation [20]. For almost the entire range of relevant sensor response, the SWC predicted by the factory calibration was considerably higher than the SWC predicted by our two-step calibration. Spelman et al. [7] reported a similar difference between the factory calibration of the 10HS sensor and soil-specific calibrations using agricultural soils.

**Figure 9:**
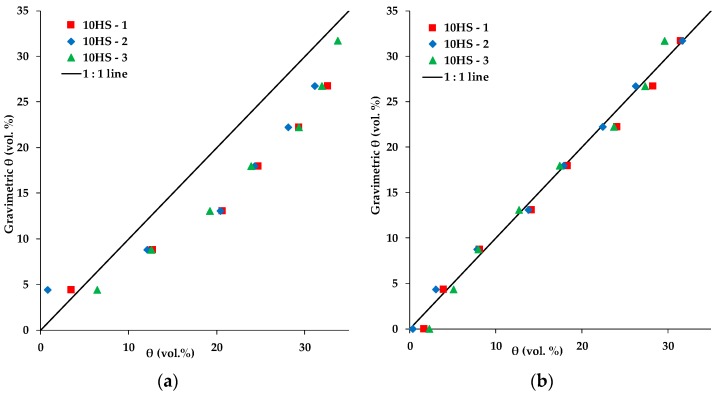
Comparison of the gravimetric soil water content with those measured with three 10HS sensors using either (**a**) the factory calibration or the (**b**) universal Sensor Response–Permittivity (SRP) model combined with the model of Topp et al. [20].

To further confirm this strong discrepancy, we conducted a sandbox experiment with three 10HS sensors. Figure 9 compares gravimetrically determined volumetric SWC with SWC determined with the 10HS sensors using the factory calibration and the universal SRP model combined with the Topp equation [20]. The factory calibration resulted in a relatively high RMSE of 5.33 vol. % (R^2^ = 0.92), whereas the two-step calibration achieved a much better agreement (RMSE: 1.03 vol. %, R^2^ = 0.99). Fares et al. [31], who studied the effect of soil organic matter on SWC measurements with 10HS sensors, obtained a similar RMSE using the factory calibration (RMSE ranged between 5.3–7.2 vol. %), but they obtained a somewhat lower accuracy for their soil-specific calibrations (RMSE ranged between 1.3–1.9 vol. %). Matula et al. [32] found similar results for various ECH_2_O sensors (5TE, EC-5, EC-10, and EC-20) using two soil media with different bulk density (average RMSE of the factory calibration was 3.3 vol. %, while average of RMSE of the soil specific calibration was 1.3 vol. %). These results confirm the accuracy of the two-step calibration approach, and highlight the limited accuracy of the factory calibration provided with the 10HS sensor.

### 3.5. Analysis of Field Measurements

**Figure 10:**
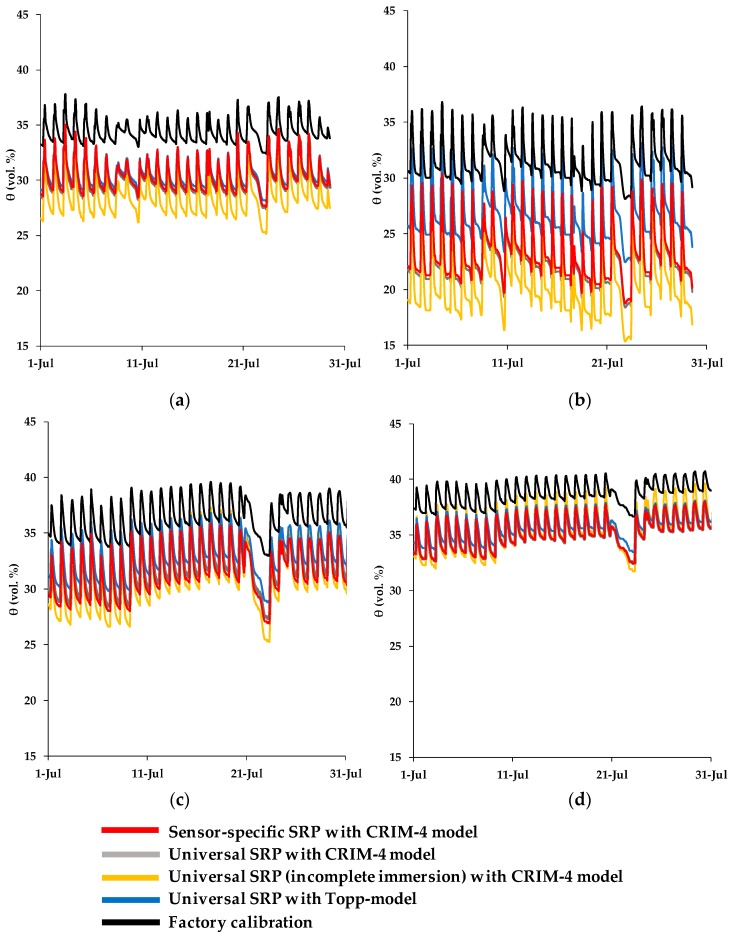
Soil water content measurements (vol. %) obtained from different calibration variants: (**a**) Menàrguens at 20 cm depth, (**b**) Menàrguens at 50 cm depth, (**c**) Mollerussa at 15 cm depth and (**d**) Mollerussa at 30 cm depth.

In a final step, we applied the factory calibration and different variants of the two-step calibration to the experimental field data used for irrigation scheduling in a period of intensive irrigation in July 2017 for both the Menàrguens and Mollerussa sites and the two measurements depths (Figure 10). The different SWC prediction variants based on the two-step calibration approach that were considered are: (i) sensor-specific SRP models combined with the CRIM-4 model, (ii) a universal SRP model combined with the CRIM-4 model, (iii) a universal SRP model obtained with incompletely immersed sensor head combined with the CRIM-4 model, (iv) and a universal SRP model combined with the Topp model [20]. It should be noted that corrections for temperature and electrical conductivity were not yet considered here, and we only focus on differences in SWC predictions using different calibration strategies.

In the following, the sensor-specific SRP models combined with the CRIM-4 model were used as a reference because this combination provided the best results for the two-step calibration approach (RMSE: 1.37 vol.%). To quantify the differences in terms of SWC predictions made with the sensor-specific SRP and the CRIM-4 model and other variants, the root mean square error (RMSE) and the mean difference were calculated (Table 9 and Table 10, respectively). The results show that the use of a universal instead of a sensor-specific SRP model in combination with the CRIM-4 model resulted in a small difference in SWC predictions with an RMSE of 0.25 vol.% and a mean difference of 0.24 vol.%. When the universal SRP model derived from calibration measurements with incompletely immersed sensor heads was used, the RMSE and the mean difference increased substantially to 1.53 vol.% and 1.31 vol.%, respectively. This increase in RMSE suggests that the effect of immersion depth is important for this particular case study. However, this is likely not generally the case, since the differences between the two immersion variants varied considerably for different sensor response ranges (see Figure 3). When the CRIM-4 model was replaced with the Topp model [20], the differences in SWC predictions resulted in a RMSE of 1.51 vol.% and a mean difference of 1.49 vol.%. Therefore, it can be concluded that the consideration of the correct immersion variant of the sensor response calibration and an accurate soil-specific calibration are more important than the use of a sensor-specific SRP model. Finally, the SWC predictions based on the factory calibration resulted in even larger differences with an RMSE of 5.18 vol.% and a mean difference of 5.16 vol.%.

The results obtained in this study can be used to improve irrigation scheduling. At the Menàrguens and Mollerussa test sites, the SWC predictions obtained by the two-step calibration approach were always below the predictions based on the factory calibration. Therefore, the factory calibration would have resulted in an underestimation of irrigation amounts for both the Menàrguens and Mollerussa sites. Although two-step calibration requires more time, it was worthwhile in this particular study, since it increased the accuracy of the SWC measurements and thus allows for a proper application of irrigation that matches the crop needs.

## 4. Conclusions

In this paper, we evaluated the sensor response of low-cost 10HS soil water content sensors using a two-step calibration procedure. First, we calibrated the sensor response of 10HS sensors to permittivity using a standard procedure based on five reference media with known dielectric permittivity. Here, the effect of immersion depth on the calibration results was also considered. Second, a site-specific relationship between permittivity and soil water content with soil samples from different sites and depths was established. It was found that the results of the calibration in reference media depended on the immersion depth of the sensor. Therefore, the calibration protocol should be adapted to the type of application of the 10HS sensor. For example, the sensor head is typically inserted into the soil in field applications. Therefore, the sensor should be calibrated with a fully immersed sensor head for this type of application. In addition, we compared the accuracy of the use of a universal calibration relationship between sensor response and permittivity with the accuracy of a sensor-specific calibration. Our results showed that the RMSE of the dielectric permittivity estimated decreased from 1.421 to 0.427 when a sensor-specific calibration was considered.

In a next step, undisturbed soil samples and time domain reflectometry (TDR) were used to establish a site-specific relationship between permittivity and soil water content. Five different model variants were used that relied on available data on porosity and fitting of the permittivity of the solid phase to a different extent. The model that considered both variations in porosity and solid-phase permittivity between sites and depths resulted in the highest accuracy (RMSE: 1.37 vol.%). However, a simplified model that considered a universal fitted value for the solid-phase permittivity and spatially variable porosity provided almost equal accuracy. Based on the two-step calibration, relationships between sensor response and soil water content were obtained that were compared to the factory calibration using measurements on sand with known water content. It was found that the relationship obtained using the two-step calibration approach provided much more accurate SWC predictions than the factory calibration provided with the 10HS sensor (RMSE: 5.33 vol.% versus 1.03 vol.%).

Finally, we applied the factory calibration and different variants of the two-step calibration approach to field measurements made with 10HS sensors during a period of irrigation in almond and apple orchards in Menàrguens and Mollerussa, respectively. The results showed that the time-average absolute difference was 5.16 vol.% and the RMSE was 5.18 vol.% when the factory calibration was used instead of the most advanced model obtained with the two-step calibration approach. The use of a universal instead of a sensor-specific sensor response model only resulted in a small difference and RMSE, thus indicating that the use of a universal sensor response model would have been possible in this particular case study. The use of the empirical equation of Topp et al. (1980) instead of a soil-specific calibration resulted in a moderate increase in the mean difference and RMSE. Since the factory calibration significantly overestimated SWC, it is recommended to improve the accuracy of SWC measurements of the 10HS sensors using sensor- and soil-specific calibration in applications where accuracy is important.

## Figures and Tables

**Table 1:** Characteristics of the soils at different deeps in almond and apple crop.

Depth (m)	Menàrguens		Mollerussa	
	0–0.5 (Ridge)	0.5–1 (Under Ridge)	0–0.2	0.2–0.4
Silt (0.002 < d < 0.05 mm) %	37.2	37.0	40.7	40.6
Clay (d < 0.002 mm) %	21.2	24.3	23.5	23.9
Sand (0.05 < d < 2 mm) %	41.6	38.7	35.8	35.5
USDA Soil Classification	Loam	Loam	Loam	Loam
Bulk density (Kg·m^−3^)	1370	1700	1480	1500

**Table 2:** Properties of the calibration media as well as the equivalent soil water content (SWC) calculated with the Topp equation [20].

Calibration Standard	Medium	Reference Permittivity	Volume Fraction i-C3E1	Volume Fraction Water	Equivalent SWC
		-	-	-	vol. %
M1	Air	1.00	-	-	-
M2	Glass beads	3.34	-	-	4.0
M3	I-C_3_E_1_/water mixture	18.14	0.92	0.08	32.0
M4	I-C_3_E_1_/water mixture	26.26	0.80	0.20	41.0
M5	I-C_3_E_1_/water mixture	34.82	0.68	0.32	48.0

**Table 3:** Properties of the samples for the topsoil and subsurface soil.

Menàrguens				Mollerussa			
Sample Name	Depth	Bulk Density	Porosity	Sample Name	Depth	Bulk Density	Porosity
	cm	g·cm^−3^	%		cm	g·cm^−3^	%
S1-Men	~20	1.40	47	S1-Moll	~15	1.56	41
S2-Men		1.33	50	S2-Moll		1.41	47
S3-Men		1.37	48	S3-Moll		1.41	47
S4-Men		1.36	49	S4-Moll		1.50	43
S5-Men	~50	1.72	35	S5-Moll	~30	1.57	41
S6-Men		1.60	40	S6-Moll *		1.47	44
S7-Men		1.75	34	S7-Moll		1.46	45
S8-Men		1.70	36	S8-Moll		1.50	43

* Sample was discarded due to shrinkage

**Table 4:** Statistical result of the sensor response measurements using 10HS sensors in calibration media.

Calibration Medium	Incompletely Immersed Sensors in Calibration Medium			Fully Immersed Sensors in Calibration Medium		
	Mean Sensor Response	Standard Deviation	Coefficient of Variation	Mean Sensor Response	Standard Deviation	Coefficient of Variation
	V	V	%	V	V	%
M1	0.50	0.009	1.84	0.50	0.009	1.84
M2	0.80	0.017	2.10	0.79	0.016	2.08
M3	1.32	0.011	0.86	1.32	0.011	0.86
M4	1.41	0.017	1.23	1.43	0.016	1.12
M5	1.47	0.015	1.01	1.52	0.017	1.10

**Table 5:** Fitting parameters and root mean square error (RMSE) between measured and predicted dielectric permittivity when the sensors are incompletely immersed and fully immersed in calibration media.

	*α*	*β*	*γ*	RMSE
Equation (2) incompletely immersed	−0.200	0.335	−1.227	0.518
Equation (2) fully immersed	−0.118	0.220	−2.456	0.412

**Table 6:** RMSE between apparent dielectric permittivity (*K_a_*) and reference permittivity for sensor-specific and universal calibration, as well as the corresponding equivalent soil water content (SWC) (*θ_eq_*) RMSE estimated using the Topp empirical permittivity-SWC relationship [20].

Calibration Standard	Sensor-Specific Calibration		Universal Calibration Function	
	RMSE *K_a_*	RMSE *θ_eq_* (vol. %)	RMSE *K_a_*	RMSE *θ_eq_* (vol. %)
M1	0.349	-	0.350	-
M2	0.398	1.014	0.426	1.083
M3	0.397	0.528	0.684	0.901
M4	0.608	0.571	1.424	1.317
M5	0.207	0.135	2.324	1.471
Total	0.427	0.642	1.421	1.213

**Table 7:** RMSE between soil water content measured and predicted by different models.

Model	RMSE (vol. %)
Topp	2.94
Complex Refractive Index Model 1 (CRIM-1)	3.54
Complex Refractive Index Model 2 (CRIM-2)	1.90
Complex Refractive Index Model 3 (CRIM-3)	1.43
Complex Refractive Index Model 4 (CRIM-4)	1.37

**Table 8:** Parameters of the complex refractive index (CRIM-4) model for Menàrguens and Mollerussa sites.

Depth	Menàrguens		Mollerussa	
	20 cm	50 cm	15 cm	30 cm
*K_water_*	78.54	78.54	78.54	78.54
*K_air_*	1.00	1.00	1.00	1.00
*K_solid_*	6.09	6.66	6.16	5.98
*η*	0.48	0.36	0.44	0.43

**Table 9:** Accuracy of the different calibration variants with respect to the reference calibration (sensor-specific Sensor Response–Permittivity (SRP) models combined with the complex refractive index (CRIM-4) model).

Calibration Variant	Menàrguens		Mollerussa		Mean RMSE (vol.%)
	20 cm	50 cm	15 cm	30 cm	
	RMSE (vol.%)				
Universal SRP with CRIM-4 model	0.17	0.32	0.50	0.02	0.25
Universal SRP (incomplete immersion) with CRIM-4 model	1.84	2.79	0.79	0.70	1.53
Universal SRP with Topp model	0.30	3.57	1.54	0.65	1.51
Factory calibration	4.09	8.23	4.90	3.51	5.18

**Table 10:** Mean difference (vol. %) between reference calibration (sensor-specific Sensor Response–Permittivity (SRP) models combined with the complex refractive index (CRIM-4) model) and different calibration variants.

Calibration Variant	Menàrguens		Mollerussa		Absolute Mean Difference (vol.%)
	20 cm	50 cm	15 cm	30 cm	
	Mean Difference (vol. %)				
Universal SRP with CRIM-4 model	−0.17	−0.32	0.50	−0.01	0.25
Universal SRP (incomplete immersion) with CRIM-4 model	−1.80	−2.73	−0.31	0.39	1.31
Universal SRP with Topp model	0.28	3.57	1.52	0.61	1.49
Factory calibration	4.07	8.21	4.87	3.49	5.16
